# Potential zoonotic sources of SARS‐CoV‐2 infections

**DOI:** 10.1111/tbed.13872

**Published:** 2020-10-23

**Authors:** Wendy K. Jo, Edmilson Ferreira de Oliveira‐Filho, Andrea Rasche, Alex D. Greenwood, Klaus Osterrieder, Jan Felix Drexler

**Affiliations:** ^1^ Institute of Virology, Charité‐Universitätsmedizin Berlin, Corporate Member of Freie Universität Berlin, Humboldt‐Universität zu Berlin, and Berlin Institute of Health Berlin Germany; ^2^ German Centre for Infection Research (DZIF) Associated Partner Charité‐Universitätsmedizin Berlin Berlin Germany; ^3^ Leibniz Institute for Zoo and Wildlife Research Berlin Germany; ^4^ Institut für Virologie Freie Universität Berlin Berlin Germany; ^5^ Martsinovsky Institute of Medical Parasitology, Tropical and Vector‐Borne Diseases Sechenov University Moscow Russia

**Keywords:** SARS‐CoV‐2, COVID‐19, coronavirus, domestic animal, carnivore, farmed animal

## Abstract

The severe acute respiratory syndrome coronavirus‐2 (SARS‐CoV‐2) causing coronavirus disease‐2019 (COVID‐19) likely has evolutionary origins in other animals than humans based on genetically related viruses existing in rhinolophid bats and pangolins. Similar to other animal coronaviruses, SARS‐CoV‐2 contains a functional furin cleavage site in its spike protein, which may broaden the SARS‐CoV‐2 host range and affect pathogenesis. Whether ongoing zoonotic infections are possible in addition to efficient human‐to‐human transmission remains unclear. In contrast, human‐to‐animal transmission can occur based on evidence provided from natural and experimental settings. Carnivores, including domestic cats, ferrets and minks, appear to be particularly susceptible to SARS‐CoV‐2 in contrast to poultry and other animals reared as livestock such as cattle and swine. Epidemiologic evidence supported by genomic sequencing corroborated mink‐to‐human transmission events in farm settings. Airborne transmission of SARS‐CoV‐2 between experimentally infected cats additionally substantiates the possibility of cat‐to‐human transmission. To evaluate the COVID‐19 risk represented by domestic and farmed carnivores, experimental assessments should include surveillance and health assessment of domestic and farmed carnivores, characterization of the immune interplay between SARS‐CoV‐2 and carnivore coronaviruses, determination of the SARS‐CoV‐2 host range beyond carnivores and identification of human risk groups such as veterinarians and farm workers. Strategies to mitigate the risk of zoonotic SARS‐CoV‐2 infections may have to be developed in a *One Health* framework and non‐pharmaceutical interventions may have to consider free‐roaming animals and the animal farming industry.

## 1 INTRODUCTION

Coronavirus disease‐2019 (COVID‐19) emerged in December 2019 in Wuhan, China and quickly spread to more than 200 countries, killing over 1 million people worldwide as of October 2020. The rapid progression of the pandemic has not only taxed healthcare systems worldwide but has also wreaked havoc on the global economy and society (UN, 2020a, 2020b). COVID‐19 is caused by a newly emerged coronavirus termed severe acute respiratory syndrome coronavirus‐2 (SARS‐CoV‐2) (Li et al., 2020). A conspecific coronavirus termed SARS‐CoV caused an outbreak in 2003–2004, infecting about 8,000 people mainly in China, of which about 10% died (Cheng et al., 2013). Control of SARS in 2003–2004 by strict non‐pharmaceutical interventions was facilitated by predominant SARS‐CoV replication in the lower respiratory tract (Gu & Korteweg, 2007), as opposed to efficient replication of SARS‐CoV‐2 in the upper respiratory tract (Wolfel et al., 2020) despite usage of the same receptor molecule angiotensin‐converting enzyme 2 (ACE2) (Hoffmann et al., 2020; Li et al., 2003). Here, we discuss the evidence for the zoonotic origins of human coronaviruses, identify epidemiologically relevant animal hosts and discuss their impact on containment strategies of COVID‐19 under a *One Health* approach, a concept that is gaining recognition which addresses health issues, such as zoonotic diseases, at the human‐animal‐environment interface in a collaborative effort among multiple relevant disciplines and sectors (FAO‐OIE‐WHO, 2019).

## 2 ZOONOTIC ORIGIN OF HUMAN CORONAVIRUSES

**Figure 1:**
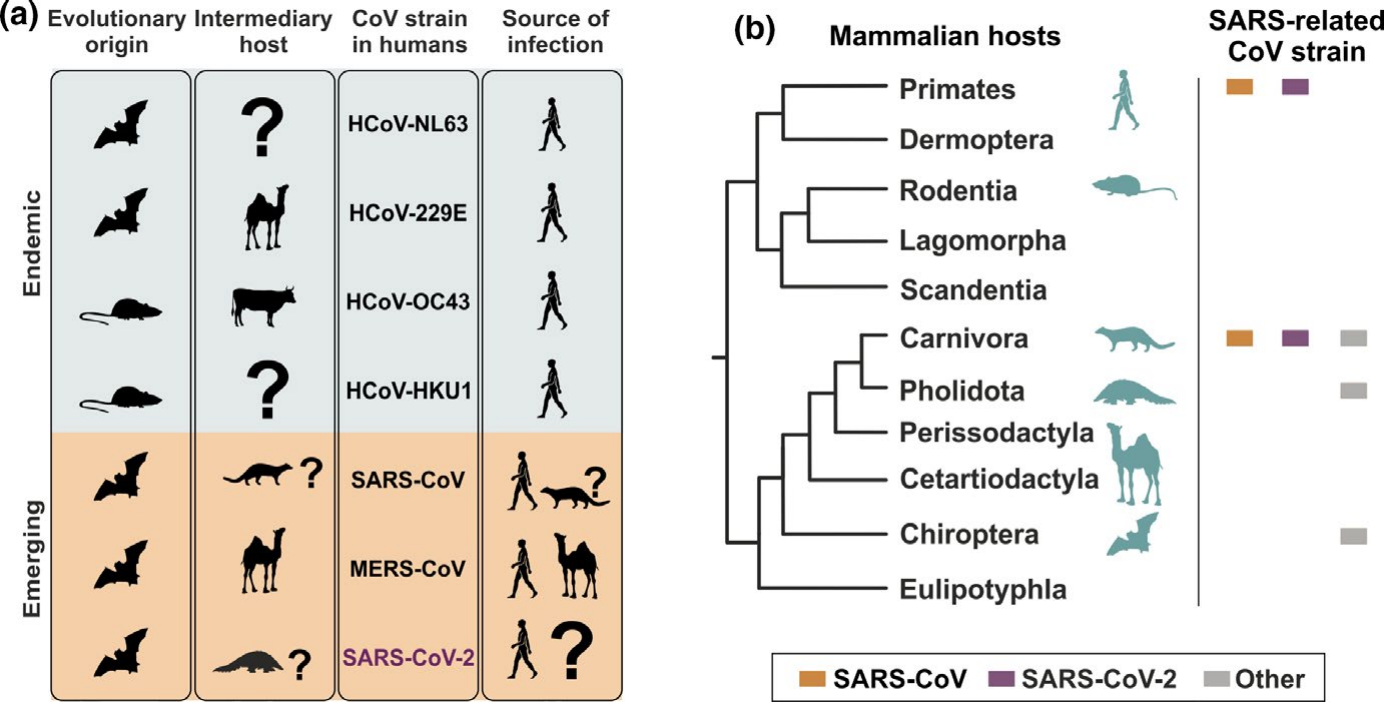
Mammals as reservoirs and intermediary hosts of endemic and emerging human coronaviruses. (a) Animal reservoirs and intermediary hosts of human coronaviruses. (b) Cladogram of mammalian orders adapted from (Foley et al., 2016). Hosts of coronaviruses are depicted by pictograms (teal). Squares depict families susceptible to SARS‐CoV (orange), SARS‐CoV‐2 (purple) and other SARS‐related CoVs (grey) [Colour figure can be viewed at wileyonlinelibrary.com]

**Figure 2:**
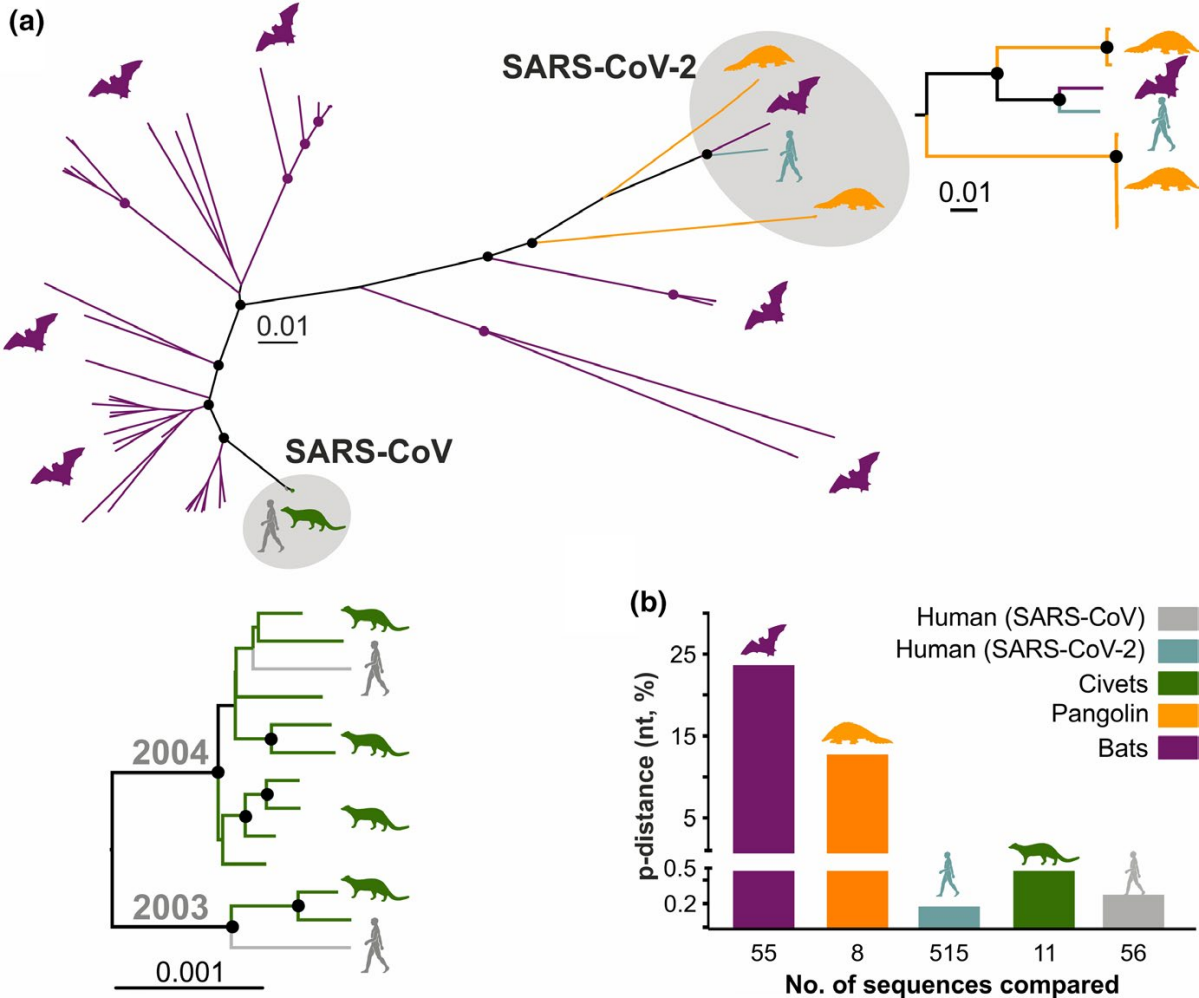
Mammalian hosts of coronaviruses. (a) Large diversity of bat‐associated SARS‐related CoVs in comparison to other hosts. The phylogeny was constructed with 77 SARS‐related CoV full‐length genomes using a neighbour‐joining method with 1,000 bootstrap replicates. Circles at nodes indicate bootstrap values ≥75%. Scale bar indicates nucleotide substitutions per site. (b) High divergence of bat SARS‐related CoVs in comparison with viruses infecting pangolin, civet and human hosts. P‐distance within SARS‐related CoVs in each host. The human SARS‐CoV‐2 and SARS‐CoV genomes included all sequences found in GenBank as of 16.04.2020, excluding identical genomes. GenBank accession numbers of all other genomes used in panel A and B: Bat SARS‐related CoVs: MN996532, MG772934, MG772933, KY938558, KU182964, KJ473811, KY770860, KJ473813, KJ473812, DQ412042, KY770859, KY770858, BKJ473816, KY417145, KF294455, DQ071615, KY417152, KY417151, KF367457, KC881006, KC881005, KY417144, KY417150, KT444582, KY417146, KY417149, KY417143, FJ588686, KY417148, KY417147, KP886809, KP886808, KJ473815, KU973692, KF569996, KJ473814, DQ648857, DQ412043, JX993987, KF294457, GQ153548, GQ153546, GQ153545, GQ153544, GQ153541, GQ153539, GQ153540, DQ084199, DQ084200, DQ022305, GQ153547, GQ153543, GQ153542, KY352407, GU190215; Human SARS‐CoV‐2: MN908947; Human SARS‐CoV: AY568539, AY390556; Civet SARS‐CoV: AY572034, AY572035, AY686863, AY686864, AY304486, AY304488, AY572038, AY686864, AY613948, AY613949, AY613950; and Pangolin SARS‐related CoVs: MT121216, MT040335, MT072865, MT072864, MT040336, MT040334, MT040333 [Colour figure can be viewed at wileyonlinelibrary.com]

Endemic human coronaviruses (HCoVs) include the alphacoronaviruses HCoV‐229E and HCoV‐NL63, as well as the betacoronaviruses HCoV‐HKU1 and HCoV‐OC43, whereas emerging HCoVs include the betacoronaviruses Middle East respiratory syndrome coronavirus (MERS‐CoV), SARS‐CoV and the novel SARS‐CoV‐2 (Figure 1a). Coronavirus species are defined based upon genomic sequence distances (Coronaviridae Study Group of the International Committee on Taxonomy of Viruses, 2020). Members of the virus species *SARS‐related coronavirus* that includes both SARS‐CoV and SARS‐CoV‐2 as well as multiple genetically highly diversified bat‐associated strains (summarized as SARS‐related CoVs henceforth) can infect different hosts belonging to four different mammalian orders, including bats, carnivores, pangolins and primates (Figure 1b). The large diversity of conspecific bat coronaviruses and their close genetic relationship to human viruses suggest that bats are potential animal reservoirs of the majority of HCoVs (Figure 2a,b) (Drexler et al., 2014). In the case of SARS‐related CoVs, rhinolophid bats (*Rhinolophus* spp., also termed horseshoe bats) are particularly relevant natural hosts, as exemplified by the extent of genetically divergent SARS‐related CoVs found in those bats across Asia, Africa and Europe (Drexler et al., 2010; Hu et al., 2017; Li et al., 2005). In the tropics, direct contact between humans and bats potentially facilitating human infection is more frequent than in temperate climates, including consumption of bats as bushmeat and their use for traditional medicine (Anti et al., 2015; Mildenstein et al., 2016). However, other animals than bats have been implicated as intermediate hosts of and source of infection with HCoVs (Figure 1a) (Corman et al., 2018). For instance, alphacoronaviruses found in camels (*Camelus dromedarius*) and alpacas (*Vicugna pacos*) share a common ancestor with HCoV‐229E (Corman et al., 2016), whereas HCoV‐OC43 shares a common ancestor with coronaviruses infecting cattle (*Bos taurus*) (Vijgen et al., 2006). The emerging MERS‐CoV is enzootic in camels (mainly *C. dromedarius*) and causes regular spill‐over infections into humans (WHO, 2020). In the case of SARS‐CoV, genetically closely related viruses were found in masked palm civets (*Paguma larvata*) and raccoon dogs (*Nyctereutes procyonoides*) sold in a live‐animal market in the Guangdong province (Guan et al., 2003; Kan et al., 2005). Genomic comparisons of full‐length SARS‐CoV sequences from human and civets showed high nucleotide identity of approximately 99.6%. In 2005, genetically highly diverse SARS‐related CoVs were found in horseshoe bats from China (Li et al., 2005). Masked palm civets were thus likely intermediate hosts, while horseshoe bats were the likely evolutionary origin of SARS‐CoV. It has been speculated that mutations in the SARS‐CoV receptor‐binding domain may have arisen in palm civets that facilitated infection efficiency by increasing the affinity to the human ACE2 receptor (Graham & Baric, 2010; Song et al., 2005).

Efforts to identify potential SARS‐CoV‐2 intermediate hosts have not been successful yet. The origin of the outbreak was associated with the Huanan seafood wholesale market in Wuhan during epidemiologic investigations of the first cases (Li et al., 2020), which was supported by SARS‐CoV‐2‐positive environmental samples from that market (Zhang & Holmes, 2020). However, retrospective epidemiologic analyses indicated that an earlier COVID‐19 case had no exposure to that seafood market (Huang et al., 2020). The origins of the pandemic thus remain unclear. The closest relatives to human SARS‐CoV‐2 known so far are a coronavirus coming from the intermediate horseshoe bat (*R. affinis*) from China with approximately 96% nucleotide identity, and two sub‐lineages of SARS‐related CoVs found in Malayan pangolins (*Manis javanica*) with 85.5%–92.4% nucleotide identity averaged over the whole viral genome (Figure 2a) (Lam et al., 2020; Xiao et al., 2020). Moreover, phylogenetic analyses using a large subgenomic data set of bat coronaviruses from China indicate that alike SARS‐CoV, SARS‐CoV‐2 likely originated in horseshoe bats (Latinne et al., 2020). However, whereas zoonotic infection of humans with civet‐associated SARS‐CoV strains was likely according to the genetic relatedness of those CoVs (Figure 2a, lower insert), bat‐, human‐ and pangolin‐associated SARS‐CoV‐2 strains are not as closely related and the direct ancestor of SARS‐CoV‐2 strains infecting humans remains to be determined (Figure 2a, right insert).

## 3 TRANSMISSION OF SARS‐COV‐2 FROM HUMANS TO ANIMALS

Human‐to‐animal transmission events during the COVID‐19 pandemic have been documented in several countries, including Hong Kong, Belgium, United States, Netherlands, Denmark, Spain, Germany and France (Barrs et al., 2020; CDC, 2020; Daly, 2020; Laudette, 2020; Oreshkova et al., 2020; ProMed‐mail, 2020a, 2020b, 2020c, 2020d; Sit et al., 2020; Sterling, 2020). Case reports on cats (*Felis catus*) living in the same household with COVID‐19 patients, revealed that these animals can be infected with SARS‐CoV‐2, showing either no or mild respiratory illness (CDC, 2020; Laudette, 2020; ProMed‐mail, 2020a, 2020c). A case report on two separate SARS‐CoV‐2‐infected dogs (*Canis lupus familiaris*), whose owners were COVID‐19 patients, showed that although both dogs tested positive by RT‐PCR and serologic methods for SARS‐CoV‐2, no apparent clinical signs were observed in the dogs (Sit et al., 2020). Other reports on SARS‐CoV‐2‐positive dogs in the Netherlands and the USA indicated that different symptoms can occur in infected dogs, ranging from mild to severe respiratory distress symptoms (Daly, 2020; Sterling, 2020). In sum, the impact of SARS‐CoV‐2 infections on domestic animal health is unclear, as both dogs and cats were reported with and without clinical signs.

A study from Wuhan showed a SARS‐CoV‐2 seroprevalence of 14.7% (15/102) in cats sampled between January and March 2020 compared with 0.0% (0/39) in cats sampled between May and March 2019 using a commercial ELISA that detects antibody reactivity against SARS‐CoV‐2 receptor‐binding domain (RBD) (Zhang et al., 2020). A study from Italy showed that 3.4% (13/388) of dogs and 3.9% (6/152) of cats living in SARS‐CoV‐2‐positive households or in regions severely affected by COVID‐19 sampled between March and May 2020, developed neutralizing antibodies against SARS‐CoV‐2 (Patterson et al., 2020). Another study, which sampled cats living in the same household with COVID‐19 patients from February to August in Hong Kong, found that 14% (6/50) were tested positive by RT‐PCR (Barrs et al., 2020). Of those positive samples, one SARS‐CoV‐2 genome was recovered and was identical to the SARS‐CoV‐2 genome recovered from the cat owner (Barrs et al., 2020). The high seroprevalence and detection rates of SARS‐CoV‐2 in cats and to some extent in dogs indicate that these animals can be infected with SARS‐CoV‐2.

Animal experimental infections can demonstrate the susceptibility of different animal species to SARS‐CoV‐2, which is on the one hand useful for the establishment of animal disease models and on the other hand provides an indication of potential animal sources that may act as animal sources of human infection. Previous infection experiments confirmed the susceptibility of ferrets (*Mustela putorius*), domestic cats and Syrian hamsters (*Mesocricetus auratus*) to SARS‐CoV (Martina et al., 2003; Roberts et al., 2005). Experimental infections of SARS‐CoV‐2 in a wide range of non‐primate animal species, including ferrets, domestic cats, raccoon dogs, Egyptian fruit bats (*Rousettus aegyptiacus*), Syrian hamsters, New Zealand white rabbits (*Oryctolagus cuniculus*) and Northern treeshrews (*Tupaia belangeris*), showed different degrees of susceptibility to the virus at inoculation doses of approximately 10^5^ 50% tissue culture infectious dose (TCID_50_) or 10^5^ plaque‐forming units (PFU) and intranasal, intratracheal and ocular transmission routes, which may be representative of natural infections during human‐to‐human transmission (Table 1) (Freuling et al., 2020; Halfmann et al., 2020; Munoz‐Fontela et al., 2020; Mykytyn et al., 2020; Schlottau et al., 2020; Shi et al., 2020; Sia et al., 2020; Zhao et al., 2020). These animals showed viral RNA shedding in the respiratory tract and to a lesser extent or no shedding in the gastroenteric tract, development of SARS‐CoV‐2‐specific antibody responses and histopathological signs of moderate inflammation in infected respiratory tissue (Freuling et al., 2020; Halfmann et al., 2020; Munoz‐Fontela et al., 2020; Mykytyn et al., 2020; Schlottau et al., 2020; Shi et al., 2020; Sia et al., 2020; Zhao et al., 2020). Notably, although Egyptian fruit bats (belonging to the family Pteropodidae, also termed pteropodid bats) are likely not a relevant host of SARS‐related CoVs in nature, their susceptibility might be due to their close genetic relationship with the likely animal reservoir of SARS‐related CoVs, namely horseshoe bats, as both pteropodid and rhinolophid bats belong to a common suborder termed Yinpterochiroptera (Teeling et al., 2005). In contrast, SARS‐CoV‐2 replicated poorly in dogs and cattle, whereas pigs (*Sus scrofa domesticus*), chickens (*Gallus gallus*) and ducks (*Anas platyrhynchos*) were not susceptible (Shi et al., 2020; Ulrich et al., 2020). Apparently, limited susceptibility of dogs in experimental infection studies contrasts the data from epidemiological studies and case reports described above. These discrepancies may be due to the different susceptibility of individual dog breeds or imperfect infection settings. In addition, airborne transmission of SARS‐CoV‐2 between cats and between hamsters has also been reported (Halfmann et al., 2020; Shi et al., 2020; Sia et al., 2020). In the case of non‐human primates, given their genetic relatedness to humans and occurrences of transmission of other human respiratory viruses such as HCoV‐OC43 (Patrono et al., 2018) and respiratory syncytial virus (Grutzmacher et al., 2016), susceptibility to SARS‐CoV‐2 was not unexpected (Munster et al., 2020; Rockx et al., 2020). Beyond their use as an animal model, human‐to‐non‐human primate transmission of SARS‐CoV‐2 may be worrying due to the possible decimation of endangered non‐human primate species in the wild. In sum, surveillance strategies should include susceptible animals in close contact to humans to prevent outbreaks of SARS‐CoV‐2 in these animals and potential spillback events to humans. Whether mutations of SARS‐CoV‐2 may arise in animals other than humans that subsequently affect pathogenesis or transmissibility in humans requires urgent investigation.

**Table 1:** Animal susceptibility to SARS‐CoV‐2

Order	Species	Mode of infection	Susceptibility	Infection dose, route	Major findings	Studies
Primates	Rhesus macaques, Cynomologous macaques	Experimental	High	4 × 10^5^–10^6^ TCID_50_ intranasal, intratracheal, ocular	Limited and moderate clinical signs, viral replication in upper and lower respiratory tracts, advanced age associated with increased histopathological changes, protective immune response	(Munster et al., 2020; Rockx et al., 2020)
Rodentia	Syrian hamster	Experimental	High	8 × 10^4^ TCID_50_, intranasal	Mild symptoms, direct contact and aerosol transmission, efficient replication in the upper and lower respiratory tract, infection of olfactory sensory neurons	(Sia et al., 2020)
Carnivora	Domestic cat	Natural and Experimental	High	1‐5.2 × 10^5^ PFU, intranasal, intratracheal, ocular	**N**: 3.4%–14.7% virus seroprevalence, 8/24 seroconversion of animals roaming around SARS‐CoV‐2‐positive mink farms, 6/50 RT‐PCR‐positive cats living in households with COVID‐19‐positive patients, no or mild symptoms **E**: aerosol transmission confirmed, lesions in nasal and tracheal mucosa epitheliums and lungs	(Barrs et al., 2020; Halfmann et al., 2020; Oreshkova et al., 2020; Patterson et al., 2020; Shi et al., 2020)
	African lion	Natural	NA	NA	Mild respiratory signs	(ProMed‐mail, 2020b)
	Malayan tiger	Natural	NA	NA	Mild respiratory signs	(ProMed‐mail, 2020b)
	Amur tiger	Natural	NA	NA	Mild respiratory signs	(ProMed‐mail, 2020b)
	Ferret	Experimental	High	10^5^ PFU, 10^5^ TCID_50_, intranasal	No or mild clinical signs, viral shedding in 8/9 animals and 3/3 contact animals, higher viral shedding in throat than rectum, histopathological changes including rhinitis and mild inflammation	(Schlottau et al., 2020; Shi et al., 2020)
	Mink	Natural	High	NA	Outbreaks in mink farms: 57 Dutch, 25 Danish, 6 USA, 1 Spain. Mild to severe respiratory symptoms, interstitial pneumonia, higher viral shedding in throat than rectum, mink‐to‐human transmission confirmed by phylogenetic analyses	(Oreshkova et al., 2020; Oude Munnink et al., 2020; ProMed‐mail, 2020d)
	Dog	Natural and Experimental	Inconclusive	10^5^ PFU, intranasal	**N**: 3.4% of animals in Italy with neutralizing animals, 2/15 RT‐PCR‐positive dogs living in households with COVID‐19‐positive patients, no to severe symptoms **E**: faecal viral shedding was detected in 3/5 inoculated dogs but no virus isolation was possible, 2/5 inoculated and 2/2 contact dogs were seronegative	(Patterson et al., 2020; Shi et al., 2020; Sit et al., 2020)
	Raccoon dog	Experimental	Moderate	10^5^ TCID_50_, intranasal	No symptoms, effective virus production and seroconversion in 6/9 animals, effective transmission to contact animals, higher viral shedding in nose and throat than rectum, mild rhinitis	(Freuling et al., 2020)
Chiroptera	Egyptian fruit bat	Experimental	High	10^5^ TCID_50_, intranasal	No symptoms, viral shedding in 9/9 inoculated animals and 2/3 contact animals, low titres of neutralizing antibodies, mild rhinitis and infiltrating lymphocytes and neutrophils	(Schlottau et al., 2020)
Artiodactyla	Cattle	Experimental	Low	10^5^ TCID_50_, intranasal	No symptoms, viral shedding and seroconversion in 2/6 animals	(Ulrich et al., 2020)
	Pig	Experimental	Negative	10^5^ PFU, 10^5^ TCID_50_, intranasal	No viral replication	(Schlottau et al., 2020; Shi et al., 2020)
Lagomorpha	New Zealand white rabbit	Experimental	Moderate	>10^5^ TCID_50_, intranasal	No symptoms, higher viral shedding in nose and throat than rectum and seroconversion in animals infected with viral dose of >10^5^ TCID50, mild histopathological changes	(Mykytyn et al., 2020)
Scandentia	Tree shrew	Experimental	Moderate	10^6^ PFU, intranasal	Increased body temperature in young animals, viral shedding in 16/24 animals, mild histopathological changes	(Zhao et al., 2020)
Galliformes	Chicken	Experimental	Negative	10^5^ PFU, 10^5^ TCID_50_, oculo‐oronasal	No viral replication	(Schlottau et al., 2020; Shi et al., 2020)
	Ducks	Experimental	Negative	10^5^ PFU	No viral replication	(Shi et al., 2020)

Abbreviations: E, Experimental; N, Natural; NA, not applicable due to limited data; PFU, Plaque forming units; TCID_50_, 50% tissue culture infectious dose.

Zoonotic SARS‐CoV‐2 transmission events are not restricted to domestic animals only. In the Bronx Zoo in New York, several felids tested positive, including two Malayan tigers (*Panthera tigris jacksoni*), two Amur tigers (*Panthera tigris altaica*) and three African lions (*Panthera leo*), all of which developed mild respiratory signs and recovered after a week. The likely source of infection was infected zoo staff (ProMed‐mail, 2020b). Similarly, SARS‐CoV‐2‐positive minks were reported in 57 mink fur farms in the Netherlands, 25 fur farms in Denmark, six fur farms in the USA and one fur farm in Spain (ProMed‐mail, 2020d). An investigation conducted in two of the Dutch farms reported that the infected minks showed mild to severe respiratory distress (Oreshkova et al., 2020). Upon post‐mortem analyses, several minks showed interstitial pneumonia (Oreshkova et al., 2020). The source of the SARS‐CoV‐2 outbreak in minks was linked to the farmers and their family members, who either showed symptoms compatible with COVID‐19 or were tested positive for SARS‐CoV‐2. Moreover, in addition to the infected minks, 8 out of 24 stray cats surrounding two of the farms showed evidence of SARS‐CoV‐2 infection, 7 of which were confirmed by a highly specific microneutralization assay and one by PCR (Oreshkova et al., 2020). Virus transmission from infected minks back to humans was corroborated by phylogenetic comparison between human‐ and mink‐derived SARS‐CoV‐2 sequences, which grouped together (Oude Munnink et al., 2020). Zoonotic infections are common occupational hazards in individuals who are frequently exposed to animals or animal products such as veterinarians, zoo and abattoir workers, breeders and farmers (Epp & Waldner, 2012). Risk assessments should be carried out to identify occupational groups that are disproportionately exposed to potentially SARS‐CoV‐2 infected animals, as was exemplified by a novel squirrel bornavirus, which caused fatal encephalitis among squirrel breeders and a zoo worker (Tappe et al., 2018).

**Figure 3:**
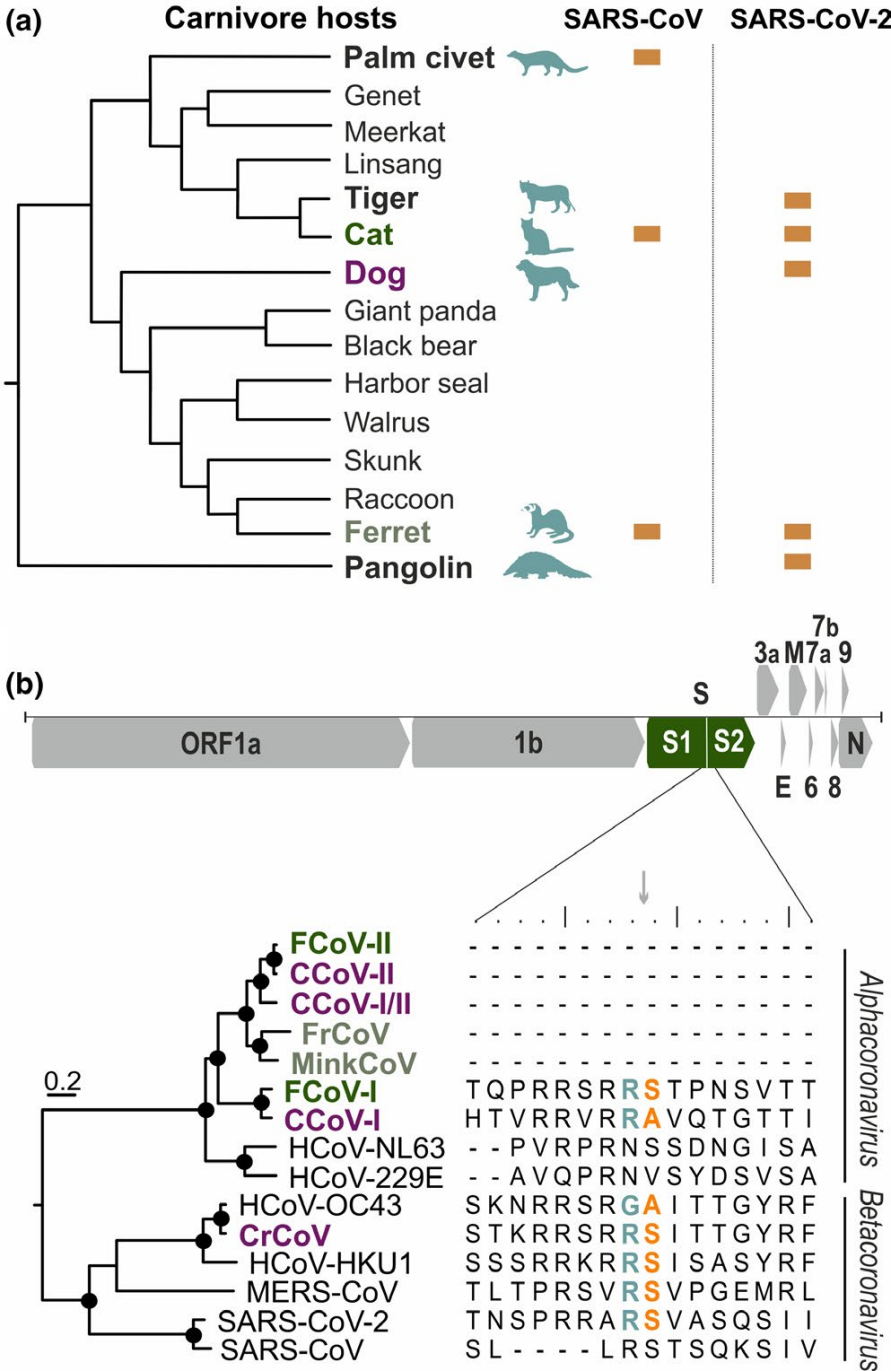
Susceptibility of carnivore hosts to coronaviruses (a) Carnivores susceptible to SARS‐related CoVs are dispersed across the family tree. Cladogram of carnivore families adapted from (Foley et al., 2016). (b) Furin cleavage site between the spike subunits S1 and S2 is predominantly present in betacoronaviruses. Scheme of SARS‐CoV‐2 genome organization with a magnified S1/S2 furin cleavage site within carnivore and human coronaviruses . Functional cleavage sites are highlighted with coloured ‘R S’ (orange and teal). Maximum‐likelihood tree of human and carnivore coronaviruses showing grouping of cat (green), dog (purple) and mustelid (grey) CoVs with human CoVs based on translated spike amino acid sequences. WAG + G + I was used as a substitution model and a complete deletion option was chosen. Scale bar indicates amino acid substitutions per site. Circle at nodes indicate bootstrap values ≥75% (1,000 bootstrap replicates). GenBank accession numbers: YP_004070194, AFG19726, AKZ66476, YP009256197, ADI80513, ACT10854, AEQ61968, YP_003767, NP_073551, AAR01015, AQT26498, YP_173238, YP_009047204, QHU36864, ACB69905 [Colour figure can be viewed at wileyonlinelibrary.com]

The combined data from natural and experimental infections together with preliminary epidemiologic investigations highlight the potential transmission of SARS‐CoV‐2 from humans to different mammalian orders, particularly members of the order Carnivora (Figure 3a, Table 1). Comparing the ACE2 residues involved in SARS‐CoV‐2 entry in humans (Shang et al., 2020) with the sequences from different carnivore species revealed only few amino acid changes, which is consistent with SARS‐CoV‐2 infection in those mammals (Table 2). It is unclear whether mutations in the SARS‐CoV‐2 spike receptor‐binding domain are needed to increase infection efficiency in different carnivore hosts or if an ACE2‐independent pathway, potentially mediated by the SARS‐CoV‐2 furin cleavage site, is employed in parallel to receptor‐mediated entry (Hoffmann et al., 2020).

**Table 2:** Critical ACE2 residues that interact with the receptor binding domain site of the SARS‐CoV‐2 spike protein based on human SARS‐CoV‐2 infection (Shang et al., 2020)

Species	ACE2 residue																				
	19	24	27	28	31	34	35	37	38	41	42	45	79	82	83	329	330	353	354	355	357
**Human**	**S**	**Q**	**T**	**F**	**K**	**H**	**E**	**E**	**D**	**Y**	**Q**	**L**	**L**	**M**	**Y**	**E**	**N**	**K**	**G**	**D**	**R**
**Domestic cat**	**S**	L	**T**	**F**	**K**	**H**	**E**	**E**	E	**Y**	**Q**	**L**	**L**	T	**Y**	**E**	**N**	**K**	**G**	**D**	**R**
**Dog**	**S**	L	**T**	**F**	**K**	Y	**E**	**E**	E	**Y**	**Q**	**L**	**L**	T	**Y**	G	**N**	**K**	**G**	**D**	**R**
**Ferret**	**S**	L	**T**	**F**	**K**	Y	**E**	**E**	E	**Y**	**Q**	**L**	H	T	**Y**	Q	**N**	**K**	R	**D**	**R**
Raccoon	**S**	L	**T**	**F**	N	N	**E**	**E**	E	**Y**	**Q**	**L**	Q	T	**Y**	**E**	**N**	**K**	**G**	**D**	**R**
Polar bear	**S**	L	**T**	**F**	**K**	Y	**E**	**E**	**D**	**Y**	**Q**	**L**	H	T	**Y**	**E**	**N**	**K**	**G**	**D**	**R**
Civet	**S**	L	**T**	**F**	T	Y	**E**	Q	E	**Y**	**Q**	V	**L**	T	**Y**	**E**	**N**	**K**	**G**	**D**	**R**
Meerkat	**S**	L	**T**	**F**	Q	**H**	**E**	Q	E	**Y**	L	V	R	A	**Y**	D	**N**	**K**	**G**	**D**	**R**
Hyena	**S**	L	**T**	**F**	**K**	Y	**E**	Q	E	**Y**	L	**L**	**L**	T	**Y**	**E**	**N**	**K**	**G**	**D**	**R**
Harbour seal	**S**	L	**T**	**F**	**K**	Y	**E**	**E**	E	**Y**	**Q**	**L**	Q	T	**Y**	**E**	**N**	**K**	R	**D**	**R**
Sea lion	**S**	L	**T**	**F**	**K**	S	**E**	**E**	E	**Y**	**Q**	**L**	Q	T	**Y**	**E**	**N**	**K**	H	**D**	**R**
Walrus	**S**	L	**T**	**F**	**K**	Y	**E**	**E**	E	**Y**	**Q**	F	Q	T	**Y**	**E**	**N**	**K**	H	**D**	**R**
Pangolin	**S**	E	**T**	**F**	**K**	S	**E**	**E**	E	**Y**	**Q**	**L**	I	N	**Y**	**E**	**N**	**K**	H	**D**	**R**
Horseshoe bat	**S**	E	M	**F**	**K**	T	K	**E**	**D**	H	**Q**	**L**	**L**	N	**Y**	N	**N**	**K**	**G**	**D**	**R**
	*		*	*	*		*	*		*	*	*			*		*	*		*	*

Note

Cat, dog and ferret residues identical to human ACE2 critical residues are highlighted with an asterisk.

## 4 FURIN CLEAVAGE SITE IN HUMAN AND CARNIVORE CORONAVIRUSES

Different from SARS‐CoV, SARS‐CoV‐2 contains a furin protease cleavage site in the viral spike protein, which is responsible for attachment and entry into cells (Coutard et al., 2020). In MERS‐CoV and influenza viruses, presence of a furin cleavage site has been associated with enhanced transmissibility, increased virulence and broader host range (Andersen et al., 2020). It has been speculated that furin‐mediated spike cleavage may also affect SARS‐CoV‐2 pathogenesis (Andersen et al., 2020). Several feline coronavirus (FCoV) strains also have a furin cleavage site in their spike proteins (Figure 3b) (Licitra et al., 2013). Interestingly, mutations in the furin cleavage site are mainly found in the feline infectious peritonitis virus (FIPV) biotype, which constitutes the virulent variant of FCoV. (Licitra et al., 2013). It is still a matter of debate if the observed mutations contribute to the acquisition of monocyte and macrophage tropism, a hallmark of FIPV (Licitra et al., 2013). SARS‐CoV‐2 studies investigating virus compartmentalization defining distinct viral populations, which may lead to different biotypes similarly to FCoV, should thus be conducted.

The bat/pangolin‐associated SARS‐CoV‐2‐related coronaviruses do not contain a functional furin cleavage site, yet a furin‐like cleavage site appears in other betacoronaviruses such as MERS‐CoV, HCoV‐HKU1 and HCoV‐OC43 (Figure 3b). The evolutionary origins of furin cleavage motifs in CoVs are not clear. Frequent recombination and high genetic diversity of bat‐associated SARS‐related CoVs (Hu et al., 2017; Latinne et al., 2020) may support an origin in the bat reservoir, but whether the motif emerged in a common coronavirus ancestor and was subsequently lost in most alphacoronaviruses, or whether the furin cleavage site was acquired independently from unknown sources remains to be determined.

## 5 CORONAVIRUSES NATURALLY INFECTING CARNIVORES

Besides from FCoV, which was already introduced in the section above, enzootic carnivore coronaviruses include canine coronavirus (CCoV), canine respiratory coronavirus (CrCoV), ferret coronavirus (FrCoV) and mink coronavirus (MinkCoV) (Figure 3b). Most of the mentioned carnivore CoVs belong to the genus *Alphacoronavirus* along with HCoV‐229E and HCoV‐NL63. CrCV is the only carnivore CoV within the genus *Betacoronavirus*, which includes the other HCoVs, namely HCoV‐OC43, HCoV‐HKU1, MERS‐CoV and SARS‐related CoV strains (Figure 3b).

Modified live attenuated FIPV and inactivated CCoV vaccines are commercially available. However, the use of FIPV vaccine has been controversial, as antibody‐dependent enhancement of infection has been reported in experimental settings, albeit not in the field (Takano et al., 2008). It is unclear what immunity to enzootic cat and dog CoVs elicited either by vaccination or natural infection will play in SARS‐CoV‐2 transmission between carnivores.

Currently, it is not known whether pre‐existing antibodies against human‐endemic CoVs afford cross‐ protection against or immune enhancement of SARS‐CoV‐2 infection. Despite decades of investigation into endemic HCoVs, no vaccine has been developed to prevent infections with HCoV‐229E, HCoV‐OC43, HCoV‐NL63 and HKU1, as these viruses mostly generate mild disease (Corman et al., 2018). For SARS‐CoV, translational research has been limited as the importance of the virus decreased due to the successful containment of the virus by timely public health interventions (Cheng et al., 2013). In contrast, several vaccine candidates have been tested against the recently emerged MERS‐CoV, including vectored vaccines based on adenovirus and poxvirus backbones (Alharbi, 2017). Because MERS‐CoV naturally infects camels, and human infections are almost exclusively zoonotic outside of outbreaks in hospital settings (Corman et al., 2018), MERS‐CoV vaccines were developed following a *One Health* approach considering applicability to both humans and camels (Alharbi, 2017). A vaccine candidate using a poxvirus vector is now in phase I trial as of December 2019 after tests in dromedary camels were successful (Alharbi et al., 2019; NLM, 2019).

Further investigation into animal coronavirus immune responses will be required, particularly into the implications of pre‐existing carnivore coronavirus immunity on SARS‐CoV‐2 infections including both cross‐protection and potential immune enhancement, which can shed light on the immune interplay in HCoV infections and potentially inform SARS‐CoV‐2 vaccine development.

## 6 CONCLUSION

Pandemic SARS‐CoV‐2 spread combined with over 800 million cats and dogs kept as pets worldwide (Bedford, 2020), and the large extent of the animal farming industry (Ritchie, 2017) raises concerns of domestic and farmed animals in general and carnivores in particular, becoming an epidemiologically relevant animal source for COVID‐19. Non‐pharmaceutical interventions to contain spread of SARS‐CoV‐2 may be compromised if (1) the close interaction between humans and companion or farmed animals contributes to human infections and if (2) infected domestic animals roam freely, potentially contributing to virus transmission between different households. If that was the case, strategies to mitigate and contain SARS‐CoV‐2 zoonotic transmission should be developed in a *One Health* framework, implementing molecular and serological surveillance as well as epidemiological assessment of SARS‐CoV‐2 occurrence in domestic and farmed animals alongside humans.

## CONFLICT OF INTEREST

Authors declare no competing interests.

## ETHICAL APPROVAL

No ethical approval was required as this is a review article with no original research data.

## Data Availability

Data sharing is not applicable as no new data was created.
